# TRPM7 Kinase Is Essential for Neutrophil Recruitment and Function *via* Regulation of Akt/mTOR Signaling

**DOI:** 10.3389/fimmu.2020.606893

**Published:** 2021-02-15

**Authors:** Wiebke Nadolni, Roland Immler, Kilian Hoelting, Marco Fraticelli, Myriam Ripphahn, Simone Rothmiller, Masayuki Matsushita, Ingrid Boekhoff, Thomas Gudermann, Markus Sperandio, Susanna Zierler

**Affiliations:** ^1^ Walther Straub Institute of Pharmacology and Toxicology, Ludwig-Maximilians-Universität München, Munich, Germany; ^2^ Walter Brendel Centre of Experimental Medicine, Biomedical Center, Institute of Cardiovascular Physiology and Pathophysiology, Ludwig-Maximilians-Universität München, Munich, Germany; ^3^ Bundeswehr Institute of Pharmacology and Toxicology, Munich, Germany; ^4^ Department of Molecular and Cellular Physiology, Graduate School of Medicine, University of the Ryukyus, Okinawa, Japan; ^5^ Institute of Pharmacology, Johannes Kepler University Linz, Linz, Austria

**Keywords:** TRPM7, ion channel, kinase, neutrophils (PMNs), innate immunity, inflammation, chemotaxis, migration

## Abstract

During inflammation, neutrophils are one of the first responding cells of innate immunity, contributing to a fast clearance of infection and return to homeostasis. However, excessive neutrophil infiltration accelerates unsolicited disproportionate inflammation for instance in autoimmune diseases such as rheumatoid arthritis. The *transient-receptor-potential* channel-kinase TRPM7 is an essential regulator of immune system homeostasis. Naïve murine T cells with genetic inactivation of the TRPM7 enzyme, due to a point mutation at the active site, are unable to differentiate into pro-inflammatory T cells, whereas regulatory T cells develop normally. Moreover, TRPM7 is vital for lipopolysaccharides (LPS)-induced activation of murine macrophages. Within this study, we show that the channel-kinase TRPM7 is functionally expressed in neutrophils and has an important impact on neutrophil recruitment during inflammation. We find that human neutrophils cannot transmigrate along a CXCL8 chemokine gradient or produce reactive oxygen species in response to gram-negative bacterial lipopolysaccharide LPS, if TRPM7 channel or kinase activity are blocked. Using a recently identified TRPM7 kinase inhibitor, TG100-115, as well as murine neutrophils with genetic ablation of the kinase activity, we confirm the importance of both TRPM7 channel and kinase function in murine neutrophil transmigration and unravel that TRPM7 kinase affects Akt1/mTOR signaling thereby regulating neutrophil transmigration and effector function. Hence, TRPM7 represents an interesting potential target to treat unwanted excessive neutrophil invasion.

## Introduction

The melastatin-like *transient-receptor-potential* (TRP) cation channel, TRPM7, is a bifunctional protein fusing a channel pore selective for divalent cations (Mg^2+^, Ca^2+^, Zn^2+^) to a C-terminal alpha-type serine/threonine kinase (1–4). TRPM7 channel and kinase activities are interdependent in that Mg^2+^ enters through the channel pore and the kinase domain requires Mg^2+^ ions for its activity (5). Thus, suppression of the channel activity might also reduce kinase function (6). Known substrates of the TRPM7 kinase include annexin A1, myosin II, Rho A, and SMAD2 (5, 7, 8). TRPM7 has been proposed to be essential for lymphocyte proliferation and development (9, 10). We recently established that TRPM7 kinase activity regulates differentiation of T lymphocytes toward pro-inflammatory T_H_17 cells, while anti-inflammatory regulatory T cells remain unaffected (7). Thereby the channel-kinase reins immune system homeostasis (5, 7, 11). Moreover, TRPM7 is indispensable for lipopolysaccharides (LPS)-induced activation of murine macrophages (12) and has previously been suggested to promote the differentiation of M1 over M2 macrophages (13). The role of TRPM7 in neutrophil function, though, remains unclear. Neutrophils are the most abundant leukocytes in blood and one of the first responding cells during acute inflammation. Therefore, they are essential players in innate immunity (14, 15). Following activation, neutrophils migrate toward the inflammatory site, where they eliminate pathogens *via* phagocytosis, thereby helping to restore tissue homeostasis. Another key function of neutrophils is the secretion of various cytokines and antimicrobial factors as well as the production of reactive oxygen species (ROS), killing invading pathogens (16). To prevent the spread of the infection, neutrophils are able to release so called neutrophil extracellular traps (NETs), which in turn contribute not only to bacterial clearance but also support blood vessel occlusion and immunothrombosis (17). It is well established that Ca^2+^ signaling is pivotal for the recruitment cascade and activation of neutrophils, highlighting the importance of ion channels for neutrophil function (18, 19). TRPM7 channel activity has been implicated as a regulator of cell migration by facilitating Ca^2+^ oscillations (20–22). Moreover, TRPM7 was suggested to be involved in neutrophil chemotaxis, adhesion and invasiveness with, yet, rather conflicting results (23–25). The precise mechanism how TRPM7 might be involved in neutrophil transmigration and activity remains to be explored. Patients with inherited neutrophil deficiencies suffer from severe infections, underscoring the importance of this cell type in immune defense (14). Moreover, unwarranted neutrophil infiltration accelerates tissue damage due to unsolicited inflammation in inflammatory disorders such as multiple sclerosis or rheumatoid arthritis (26, 27). Thus, it is critical to gain a better understanding of the role of TRPM7 channel and kinase activities in the signaling cascades triggering neutrophil recruitment.

Using pharmacologic blockade of TRPM7 channel or kinase as well as genetic inhibition of TRPM7 kinase activity, we here resolve the indispensable role of TRPM7 in human and murine neutrophil transmigration and production of reactive oxygen species. We further identify a potential underlying molecular mechanism, with Akt1 as novel downstream target of the TRPM7 kinase.

## Material and Methods

### Mice and *In Vivo* Experiment

*Trpm7tm1.1Mkma C56BL/6 (Trpm7^R/R^)* mice (28) were obtained from RIKEN, Japan and housed in single ventilated cages at the animal facility of the Walther Straub Institute of Pharmacology and Toxicology, Ludwig-Maximilians-Universität München, Munich, Germany. All animal experiments were performed in accordance with the EU Animal Welfare Act and were approved by the District Government of Upper Bavaria, Germany, on animal care (permit no. 55.2-1-54−2532–163–2015). Six- to twelve-week-old male and female mice were used for all experiments.

For TNF-α induced peritonitis *Trpm7^R/R^* and littermate control animals were injected i.p. with 0.9% NaCl (unstimulated) or TNF-α (500 ng, R&D Systems) and sacrificed 4 h later. Peritoneal lavage was performed using ice cold PBS and the number of extravasated neutrophils was evaluated by flow cytometry (CytoFlex S, Beckmann Coulter) and analyzed using FlowJo software. Flow-Count Fluorospheres (Beckman Coulter, Brea, USA) were added to each sample to accurately count the cells and to calculate the total number of transmigrated neutrophils (**Figure S1**). Neutrophils were defined as CD11b^+^, Ly6G^+^ population (rat anti mouse CD11b, clone M1/70, rat anti-Ly6G, clone 1A8, all 5µg/ml, BioLegend).

### Neutrophil Isolation

Human neutrophils were extracted from whole blood of healthy volunteer blood donors using Polymorphprep (AXIS-SHIELD PoC AS) or EasySep™ Direct Human Neutrophil Isolation Kit (STEMCELL Technologies Inc.). Blood sampling was approved by the ethic committee from the Ludwig-Maximilians-Universität München (Az. 611-15).

Bone marrow (BM) derived murine neutrophils were isolated from the femurs and tibiae of *Trpm7^R/R^* and control littermates in the C57BL/6J background. Murine neutrophils were purified from BM using EasySep™ Mouse Neutrophil Enrichment Kit (STEMCELL Technologies Inc.).

After isolation, cells were resuspended in HBSS buffer [containing 0.1% of glucose, 1 mM CaCl_2_, 1 mM MgCl_2_, 0.25% BSA, and 10 mM HEPES (Sigma-Aldrich), pH 7.4].

White blood cell counts were analyzed from whole blood of *Trpm7^R/R^* and control mice using a hematology analyzer (IDEXX ProCyte Dx).

### Electrophysiology

Human and murine neutrophils were isolated as described above and verified *via* Alexa Fluor^®^ 488 anti-human CD16 or PE anti-mouse Ly6G antibody staining (clone: 3G8, BioLegend, 5µg/ml), respectively, (clone: 1A8, BioLegend, 10µg/ml) using an inverted AxioVert Microscope and Zen software (Zeiss). Patch-clamp experiments in whole-cell configuration were performed as follows: currents were elicited by a ramp protocol from –100 mV to  + 100 mV over 50 ms acquired at 0.5 Hz and a holding potential of 0 mV. Inward current amplitudes were extracted at –80 mV, outward currents at +80 mV and plotted *versus* time in seconds (s). Initial capacitative currents were subtracted and data were normalized to cell size as pA/pF. Capacitance was measured using the automated capacitance cancellation function of the EPC-10 (HEKA, Lambrecht, Germany). Values over time were normalized to the cell size measured immediately after whole-cell break-in. Nominally Mg^2+^-free extracellular solution contained (in mM): 140 NaCl, 3 CaCl2, 2.8 KCl, 10 HEPES-NaOH, 11 Gluc (pH 7.2, 300 mOsm). Standard intracellular solution contained (in mM): 120 Cs-glutamate, 8 NaCl, 10 HEPES, 10 Cs-EGTA, 5 EDTA (pH 7.2, 300 mOsm). Solutions were adjusted to 300 mOsm using a Vapro 5520 osmometer (Wescor Inc). NS8593 (30 µM, Alomone labs) was added to the bath solution at least 15 min prior to electrophysiological recordings. TG100-115 {[3-(2,4-diamino-6-(3-hydroxyphenyl)pteridin-7-yl)phenol]; 20 µM, Selleckchem} was added to the bath solution at least 30 min prior to electrophysiological recordings.

### Transwell Assay

Human neutrophils were isolated using Polymorphprep, resuspended in HBSS and incubated for 30 min at 37°C with either DMSO (Ctrl.), NS8593 (30 µM), TG (20 µM), or a combination of IPI-549 (160 nM, Selleckchem) and Nemiralisib (100 nM, Selleckchem), respectively. 3x10^5^ cells were then added to the upper compartment of the transwell filters (5 µm pore size, Corning) placed in a 24 well plate and allowed to migrate toward CXCL8 gradient in the lower compartment (10nM in HBSS, Peprotech) for 45 min at 37°C. HBSS alone was used as a negative control (unstimulated). The transwell assay was also performed using isolated murine neutrophils. Cells were incubated with DMSO (Ctrl) or NS8593 (30 µM) for 30 min at 37°C and CXCL1 (10 nM, PeproTech) was used as chemoattractant. Migrated cells were collected, stained for CD15 (clone HI98) and CD66b (clone G10F5, human) or against Ly6G (clone 1A8, murine, all 5 μg/ml, BioLegend), respectively. Cell numbers were quantified by flow cytometry (CytoFlex S, Beckmann Coulter) and Flow-Count Fluorospheres (Beckman Coulter, Brea, USA). FlowJo software was used to analyze flow cytometry data.

### Phagocytosis Assay

Heparinized human whole blood was pre-treated with DMSO, NS8593 (30 µM), TG100-115 (20 µM), or a combination of IPI-549 (160 nM) and nemiralisib (100 nM), respectively for 30 min at 37°C and then incubated with fluorescent *Escherichia coli* bio particles (pHrodo green *E. coli* BioParticle Phagocytosis Kit for flow cytometry, Thermo Fisher Scientific) for 30 min at 37°C. Phagocytosis was stopped and cells were fixed according to the manufacturer’s protocol. As control, whole blood was incubated with the particles for 30 min at 4°C. Samples were analyzed by flow cytometry (CytoFlex S, Beckmann Coulter) and FlowJo software. CD15^+^/CD66b^+^ populations were defined as neutrophils.

### 2’,7’-Dichlorofluorescin Diacetate Assay

For measurement of cellular reactive oxygen species (ROS) in human neutrophils 2’,7’-dichlorofluorescin diacetate assay (DCFDA) Cellular ROS Detection Assay Kit (Abcam, catalog# ab113851) was used according to the manufacturer’s instructions. Cells were stained and afterward preincubated with DMSO, NS8593 (30 µM, Alomone labs), TG100-115 (20 µM, Selleckchem) or a combination of IPI-549 (160 nM, Selleckchem) and Nemiralisib (100 nM, Selleckchem) for 30 min. Cells were then stimulated with LPS (10 ng/ml) for 15, 30, 60, and 90 min. Fluorescence was detected with a PolarStar Omega (BMG LABTECH) plate reader. After background subtraction ratio of relative fluorescence intensity of control and treated cell was calculated.

### Bio-Plex Assay

For Bio-Plex Pro™ Cell Signaling Assay (Bio-Rad) human and murine neutrophils were pre-incubated with DMSO, NS8593 (30 µM, Alomone Labs), TG100-115 (20 µM, Selleckchem) or a combination of IPI-549 (160 nM, Selleckchem) and nemiralisib (100 nM, Selleckchem) for 30 min and then treated with LPS (10 ng/ml, Sigma-Aldrich) for 30 min at 37°C. Cells were then washed and lysed using Cell Signaling Reagent Kit (Bio-Rad, catalog #171-304006M). Protein amount was measured using *DC*™ protein assay kit II (Bio-Rad, catalog #500-0012). Afterward samples were stored at −80°C. Collected samples were processed for the assay according to manufacturer’s instructions with following phosphoprotein targets: Akt (Ser^473^, catalog #171-V50001M), Erk1/2 (Thr^202^/Tyr^204^, Thr^185^/Tyr^187^, catalog #171-V50006M), NF-κB p65 (Ser^536^, catalog #171-V50013M), mTOR (Ser^2448^, catalog #171-V50033M).

### Statistical Analysis

All experimental data are presented as mean ± standard error of the mean (s.e.m.). Statistical analyses were performed using GraphPad Prism 8 (GraphPad Software, LLC). Comparisons between two groups were carried out using Student’s *t*-test. Differences between more than two groups were compared either by one-way or two-way analysis of variance (ANOVA), respectively, and Sidak’s multiple comparison test was used as a *post-hoc* test. Each experiment was performed independently at least in triplicate.

## Results

### TRPM7 Is Essential for Human Neutrophil Chemotaxis and ROS Production

**Figure 1:**
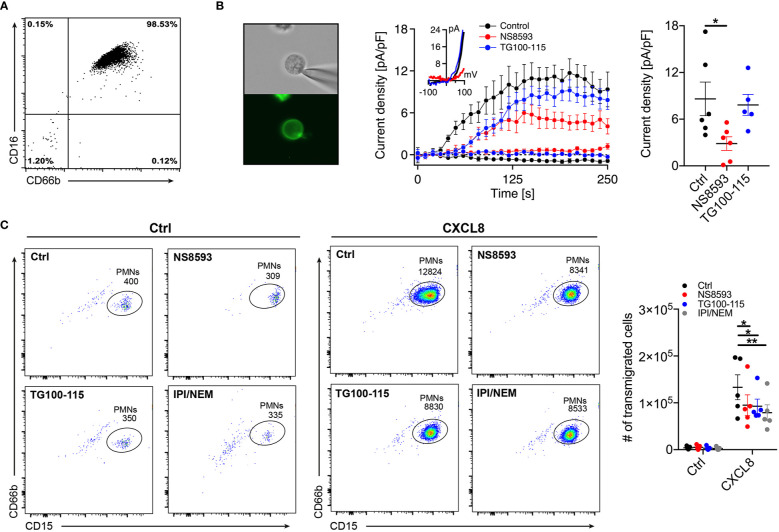
TRPM7 is essential for human neutrophil transmigration. **(A)** Representative purity of primary human neutrophils isolated from whole blood using magnetic cell sorting. **(B)** Representative human neutrophils stained with Alexa Fluor-488 conjugated anti-CD16 antibody (left panel). Whole-cell patch clamp analysis of TRPM7 ion channel activity. TRPM7 current densities in human neutrophils treated with 30 µM NS8593 (NS8593, red circles, *n* = 6), 20 µM TG100-115 (TG, blue circles, n = 5), or without (Ctrl, black circles, *n* = 6) were plotted *versus* time of the experiment in seconds (s). Error bars indicate s.e.m. Representative current-voltage relationships extracted at 250 s of human neutrophils treated with NS8593 (red), TG100-115 (blue) or without (black) (middle panel). Quantification of the current density extracted at +80 mV and displayed as pA/pF at 250 s of human neutrophils treated with NS8593 (NS8593, red, *n* = 6), 20 µM TG100-115 (TG, blue, n = 5), or without (Ctrl, black, *n* = 7) (right panel). Data are shown as mean ± s.e.m. *p < 0.05, one-way ANOVA. **(C)** Representative dot plot analysis (left and middle panel) and quantification (right panel) of neutrophil chemotaxis toward a CXCL8 gradient (10 nM) in a transwell assay. Human neutrophils were pretreated with NS8593 (30 µM, red), TG100-115 (20 µM, blue), a combination of IPI-549 and nemiralisib (IPI/NEM, 160/100 nM, gray) or dissolvent (Ctrl, black), for 30 min prior to CXCL8 or saline (Ctrl) exposure. Two-way repeated measurements ANOVA, Sidak’s multiple comparison, *p < 0.05, **p < 0.01.

To validate functional expression of TRPM7 in primary human neutrophils isolated from whole blood of healthy donors (**Figure 1A**), we subjected CD16^+^ neutrophils to whole-cell patch-clamp recordings (**Figure 1B**, left panel). We confirmed TRPM7-like current activity upon depletion of intracellular magnesium (Mg^2+^) and Mg-ATP (**Figure 1B**, middle and right panel), using our standard internal and external solutions (see *Methods*). TRPM7 currents were inhibited by the pre-incubation of cells with the known TRPM7 channel inhibitor NS8593 (30 µM) for 15 min, further confirming TRPM7 channel activity in primary human neutrophils (**Figure 1B**, red trace). In contrast, the recently described TRPM7 kinase inhibitor, TG100-115 (29) (20 µM, 30 min pre-incubation), did not affect channel activity in our patch-clamp recordings (**Figure 1B**, blue trace).

To investigate whether TRPM7 is involved in the regulation of neutrophil function, we first analyzed chemotactic properties of isolated human neutrophils in a transwell assay in response to saline (**Figure 1C**, Ctrl, left panels) or the chemokine interleukin 8 (CXCL8 (30, 31),) (**Figure 1C**, middle panel, 10 nM, 45 min) by flow cytometry. Inhibition of TRPM7 channel activity, using NS8593 (30 µM, 30 min pre-incubation), resulted in significantly reduced neutrophil numbers migrating toward a CXCL8 gradient compared to controls (**Figure 1C**, right panel). Interestingly, TRPM7 kinase blockade, using TG100-115 (20 µM, 30 min pre-incubation), ensued a significant reduction in neutrophil CXCL8-triggered chemotaxis as well. Originally, TG100-115 was identified as potent inhibitor of phospoinositol-3-kinases (PI3K), with particular affinity to PI3K-γ and -δ isoforms. Therefore, to account for its off-target effects *via* PI3K, we added respective controls IPI-549 (anti PI3K-γ) and Nemiralisib (anti PI3K-δ) (IPI/NEM, 160 nM/100 nM, 30 min pre-incubation) (**Figure 1C**, right panel). Notably, also inhibition of PI3K-γ and -δ isoforms resulted in significantly reduced chemotactic properties. These results suggest that TRPM7 and PI3 kinases contribute to chemotaxis with a yet undefined role of TRPM7 kinase activity.

**Figure 2:**
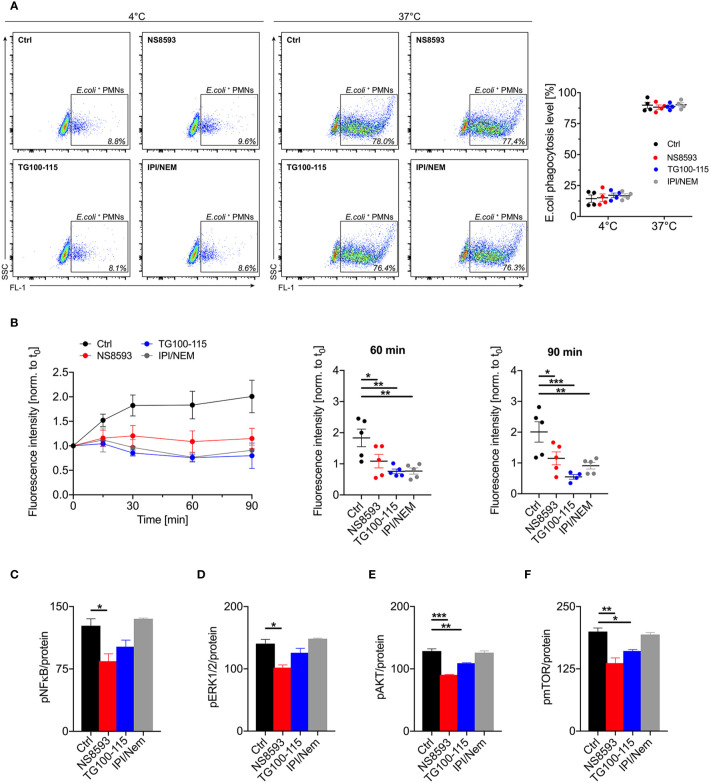
TRPM7 activity is dispensable for phagocytosis but indispensable for reactive oxygen species (ROS) production of human neutrophils. **(A)** Phagocytic activity of neutrophils was measured using fluorescent *Escherichia coli* particles together with human whole blood pre-incubated with NS8593 (30 µM, red), TG100-115 (20 µM, blue), or a combination of IPI-549 and nemiralisib (IPI/NEM, 160/100 nM, gray) for 30 min or vehicle (Ctrl, black) and analyzed by flow cytometry (n = 5). Representative dot plot analysis (left panel) and quantification of phagocytic activity (right panel). Data are shown as mean ± s.e.m., two-way repeated measurements ANOVA, Sidak’s multiple comparison. **(B)** Effects of TRPM7 channel and kinase blockade on lipopolysaccharides (LPS)-triggered ROS production. Human neutrophils were pretreated with or without (Ctrl, black), NS8593 (30 µM, red), TG100-115 (20 µM, blue), or a combination of IPI-549 and nemiralisib (IPI/NEM, 160/100 nM, gray) for 30 min and then incubated with LPS (10 ng/ml) for 0, 15, 30, 60 and 90 min. Intracellular ROS levels over time (left panel) and quantification at 60 min (middle panel) and 90 min (right panel). Data are normalized to t**_0_** and represented as mean ± s.e.m.; n=5. Statistics: one-way ANOVA *p < 0.05, ** p < 0.01, ***p < 0.005. **(C–F)** Assessment of the activity of the cell signaling molecules NFκB, Erk1/2, Akt1, and mTOR exploiting a Bio-Plex assay and phospho-specific antibodies. Primary human neutrophils were pre-incubated with or without (Ctrl, black) the TRPM7 inhibitor NS8593 (30 µM, red), the TRPM7 kinase blocker TG100-115 (20 µM, blue), or a combination of IPI and NEM (160 and 100 nM, gray), respectively, for 30 min prior to stimulation with 10 ng/ml LPS. Phosphorylation status of human neutrophils upon stimulation with 10 ng/ml LPS for 30 min of **(A)** NFκB p65 (Ser536), **(B)** Erk1/2 (Thr202/Tyr204, Thr185/Tyr187), **(C)** Akt (Ser473), and **(D)** mTOR (Ser2448), were analyzed. Data were normalized to protein content and presented as mean ± s.e.m.; biological n = 3 and measured in duplicates. Statistics: one-way ANOVA *p < 0.05, **p < 0.01, ***p < 0.005.

Another important physiological function of neutrophils is the killing of invading pathogens *via* phagocytosis and reactive oxygen burst. In order to elucidate whether this functionality is also affected by TRPM7 blockade, we evaluated phagocytic activity and ROS production of human neutrophils in the presence of TRPM7 inhibitors. To test for phagocytic activity, we incubated human whole blood from healthy donors with fluorescent *E. coli* particles and analyzed phagocytosis by flow cytometry. While phagocytosis was not affected by TRPM7 channel blockade (NS8593, 30 µM) or kinase inhibition using TG100-115 (20 µM, blue), or a combination of IPI-549 and nemiralisib (IPI/NEM, 160/100 nM, gray) (**Figure 2A**), we found differences in ROS production in response to the bacterial lipopolysaccharid LPS. TRPM7 channel blockade using NS8593 (30 µM) and TRPM7 kinase blockade using TG100-115 (20 µM) resulted in significantly less ROS production upon stimulation in human neutrophils compared to control cells (**Figure 2B**). Comparable to chemotaxis, ROS production was also affected in neutrophils by the inhibition of PI3K-γ and -δ isoforms demonstrating that TRPM7 and PI3K-γ and -δ isoforms mediate similar neutrophil functions suggesting that TRPM7 and PI3K-γ and -δ isoforms might follow analogous or even sequential signaling pathways.

### TRPM7 Regulates Human Neutrophil Function *via* NFκB, Erk, and Akt/mTOR Signaling Pathways

To further unravel the mechanism and signaling pathways by which TRPM7 channel and/or kinase blockade alters neutrophil function, we analyzed the effect of the different TRPM7 moieties on cellular signaling. Typically, chemotaxis and migration of neutrophils is regulated by LPS-triggered pathways followed by NFκB activation (32, 33). For cell motility and chemotaxis also Erk1-dependent signaling cascades are essential (34). Aside of NFκB and Erk1, PI3K/Akt/mTor signaling pathways regulate neutrophil function and recruitment (35, 36). Therefore, we employed a bead-based Bio-Plex assay (Bio-Rad) to simultaneously measure the phosphorylation status of multiple signaling proteins in the same sample (37). We examined phosphorylation levels of NFκB (p65 Ser^536^), ERK1/2 (Thr^202^/Tyr^204^, Thr^185^/Tyr^187^), Akt (Ser^473^), and mTOR (Ser^2448^) in LPS stimulated primary human neutrophils (**Figures 2C–F**). TRPM7 channel blockade using NS8593 (30 µM) revealed a reduction in NFκB, ERK1, and Akt/mTOR-dependent signaling, whereas TRPM7 kinase blockade using TG100-115 (20 µM) only led to a reduction in Akt-dependent signaling. Interestingly, inhibition of PI3K-γ and -δ using IPI-549 and Nemiralisib (IPI/NEM, 160 nM/100 nM) did not show any impact (**Figures 2C–F**) on the phosphorylation status of the detected proteins in human neutrophils. This indicates that TRPM7 might not directly signal through activation of PI3K-γ and -δ in human neutrophils or that other isoforms can compensate, at least for the investigated downstream signaling molecules. The impact of PI3K-γ and -δ blockade on chemotaxis and ROS production might not involve the analyzed signaling targets and thus might not be compensated for.

### TRPM7 Kinase Is Essential for Neutrophil Transmigration and Infiltration in an *In Vivo* Peritonitis Model

**Figure 3:**
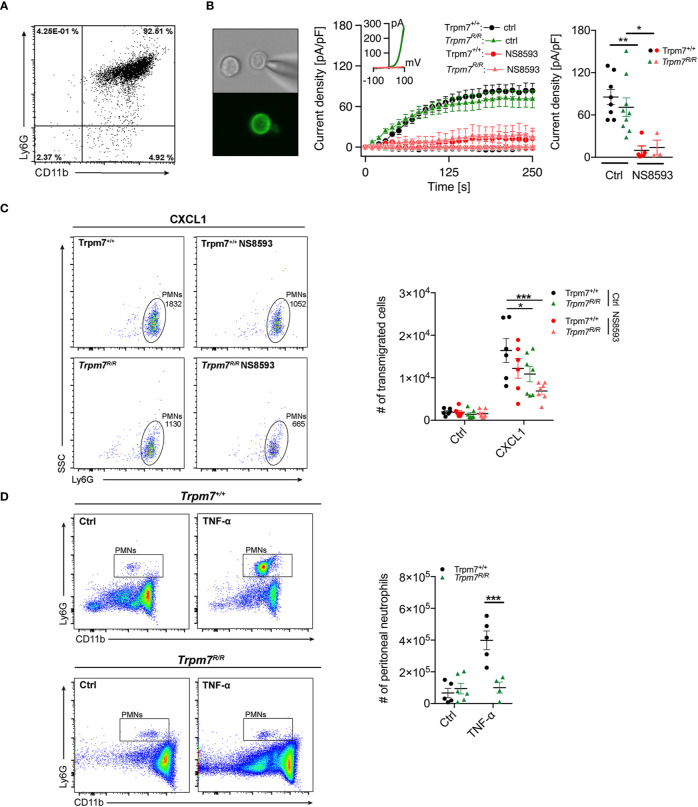
TRPM7 kinase is essential for neutrophil chemotaxis and infiltration in an *in vivo* murine peritonitis model. **(A)** Representative purity of primary bone marrow derived murine neutrophils isolated from Trpm7^+/+^ and *Trpm7^R/R^* mice using magnetic cell sorting. **(B)** Representative murine neutrophil stained with PE conjugated anti-Ly6G antibody (left panel). Whole-cell patch clamp analysis of TRPM7 ion channel activity. Primary murine neutrophils were treated with or without NS8593 (30 µM). TRPM7 current densities in neutrophils isolated from Trpm7^+/+^ mice (circles) without (black, n = 8) and with NS8593 treatment (red, n = 5) as well as from *Trpm7^R/R^* mice (triangles) without (green, n = 9) or with NS8593 (red, n = 3) were averaged and plotted *versus* time of the experiment in seconds (s) (left panel). Error bars indicate s.e.m. Representative current-voltage relationships extracted at 250 s of murine neutrophils (middle panel). Quantification of the current density extracted at +80 mV and displayed as average current density (pA/pF) at 250 s (right panels). Data are shown as mean ± s.e.m. Statistics: one-way ANOVA *p < 0.05, **p < 0.01. Note: there is no difference in TRPM7 current development or amplitude between the two genotypes. **(C)** Representative dot plot analysis (left and middle panel) and quantification (right panel) of murine neutrophil chemotaxis toward a CXCL1 gradient (10 nM) in a transwell assay. Two-way repeated measurements ANOVA, Sidak’s multiple comparison, *p < 0.05, ***p < 0.001. **(D)** Murine TNF-α peritonitis model. Saline (Ctrl), or TNF-α (500 ng) were injected intra-peritoneally into Trpm7^+/+^ (black) and *Trpm7^R/R^* (green) mice. Numbers of recruited neutrophils in the peritoneum were assessed 4 h later (n = 4–6 mice per group) using flow cytometry. Representative dot plot analyses of recruited neutrophils (left panel) and quantification (right panel). Data are shown as mean ± s.e.m. Statistics: two-way ANOVA, Sidak’s multiple comparison. ***p ≤ 0.001.

To further elucidate the role of TRPM7 kinase activity for neutrophil function, we next analyzed whether genetic inactivation of the TRPM7 kinase conveys similar effects on neutrophil chemotaxis and transmigration as pharmacologic blockade by TG100-115. We took advantage of a mouse model carrying a point mutation at the active site of the enzyme. Mutating lysine at position 1646 to arginine (*Trpm7^R/R^*) disrupts ATP binding and thereby kinase activity (28). We have previously shown, that this mutation affectively abolishes phosphotransferase activity, while leaving the channel function of TRPM7 intact (7). Here, analogously to human neutophils, we subjected CD16^+^ bone marrow derived murine neutrophils (**Figure 3A**) from Trpm7^+/+^ and *Trpm7^R/R^* mice to whole-cell patch-clamp recordings (**Figure 3B**). We confirmed TRPM7-like current activity upon depletion of intracellular Mg^2+^ and Mg-ATP (**Figure 3B**, middle and right panel), using our standard internal and external solutions (see *Methods*). TRPM7-like currents were blocked by the application of the known TRPM7 channel inhibitor NS8593 (30 µM, pre-incubation for 15 min), further confirming TRPM7 channel activity in primary murine neutrophils (**Figure 3B**, red traces). Thus, our electrophysiologic analysis established the functional expression of TRPM7 on murine bone marrow derived neutrophils, with similar current development and amplitude as well as inhibition in response to NS8593 between the two genotypes (**Figure 3B**). This further indicates that in neutrophils the channel function is independent of its kinase activity.

To further delineate whether neutrophil transmigration is regulated by TRPM7 channel and/or kinase activities, we perfomed a transmigration-assay using primary bone marrow-derived neutrophils isolated from TRPM7 kinase-deficient (*Trpm7^R/R^*) and recpective wild-type (TRPM7^+/+^) mice. We analyzed chemotactic properties of murine neutrophils in a transwell assay (**Figure 3C**) in response to saline or CXCL1 (keratinocytes-derived chemokine, KC), the murine counterpart to CXCL8 (**Figure 3C**, left panel, 10 nM, 45 min) by flow cytometry. Inhibition of TRPM7 channel activity, using NS8593 (30 µM, 30 min pre-incubation), resulted in reduced Trpm7^+/+^ neutrophil numbers migrating toward a CXCL1 gradient compared to controls, albeit not significantly (**Figure 3C**, right panel). Interestingly, TRPM7 kinase-deficient *Trpm7^R/R^* neutrophils ensued a significant reduction in neutrophil CXCL1-triggered chemotaxis. Additional inhibition of TRPM7 channel activity using NS8593 even further reduced the numbers of migrating *Trpm7^R/R^* neutrophils in response to CXCL1. These results suggest that both TRPM7 kinase and channel activity contribute to chemotaxis of murine neutrophils.

In order to apprehend whether circulating neutrophils or other leukocyte numbers were different in *Trpm7^R/R^* mice compared to Trpm7^+/+^, we analyzed the distribution of white blood cells in whole blood performing a differential blood count *via* a hematology analyzer (IDEXX ProCyte Dx). Notably, white blood cell counts were similar between *Trpm7^R/R^* mice and Trpm7^+/+^ controls (**Figure S1A**), indicating no major differences in the composition of circulating leukocytes. To finally understand the impact of TRPM7 kinase moiety on the function of neutrophils *in vivo*, we employed a TNF-α–induced peritonitis model using TRPM7 kinase-deficient (*Trpm7^R/R^*) mice. The intraperitoneal application of TNF-α triggers the recruitment of circulating leukocytes to sites of inflammation through upregulation of endothelial-specific adhesion relevant molecules (38). We pre-treated Trpm7^+/+^ or *Trpm7^R/R^* mice with saline only (Ctrl) or TNF-α (500 ng) intraperitoneally (i.p.), respectively. Four hours post injection, we analyzed neutrophil infiltration into the peritoneal cavity *via* flow cytometry (**Figure 3D**, left panels). In Trpm7^+/+^ mice, i.p. injection of TNF-α dramatically increased the number of neutrophils in the peritoneal cavity compared to control mice (**Figure 3D**, right panels). In contrast, neutrophils from *Trpm7^R/R^* mice showed a dramatic reduction in neutrophil transmigration into the peritoneal cavity upon TNF-α stimulation (**Figure 3D** **)**, suggesting a critical role of TRPM7 kinase in this process.

### TRPM7 Kinase Regulates Neutrophil Function *via* Activation of Akt/mTOR Pathways

**Figure 4:**
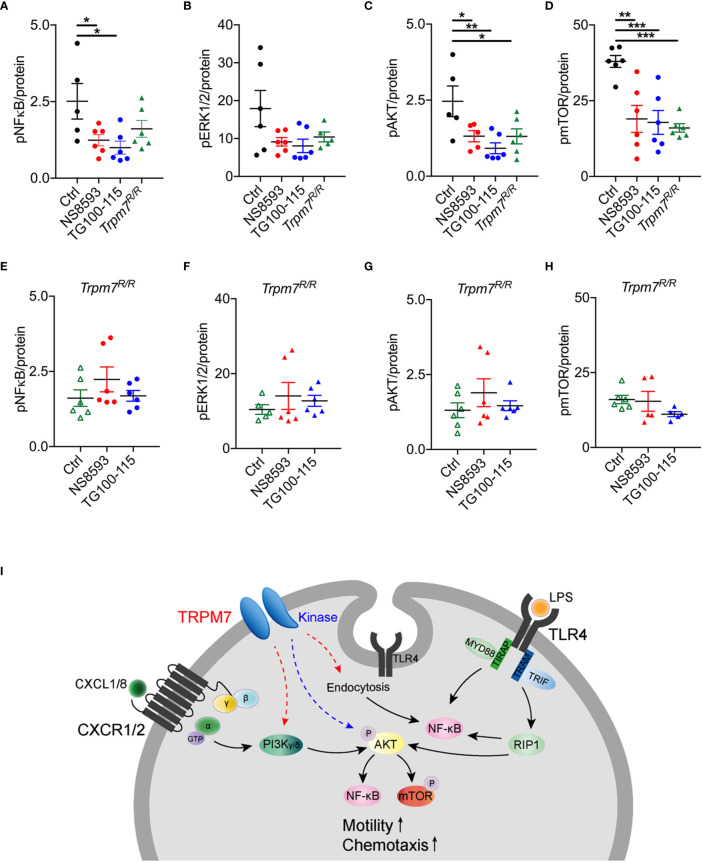
TRPM7 regulates neutrophil function *via* NFκB and Akt/mTOR signaling pathways. Assessment of the activity of the cell signaling molecules NFκB, Erk1/2, Akt1, and mTOR utilizing a Bio-Plex assay and phospho-specific antibodies on lysates of bone marrow derived murine neutrophils of Trpm7^+/+^ (black) and *Trpm7^R/R^* (green) mice. Trpm7^+/+^ and *Trpm7^R/R^* neutrophils were pre-incubated with or without (control, black) the TRPM7 inhibitor NS8593 (30 µM, red), the TRPM7 kinase blocker TG100-115 (20 µM, blue), or a combination of IPI and NEM (160 and 100 nM, gray), respectively, for 30 min. Presented data depict the phosphorylation status upon stimulation with 10 ng/ml LPS for 30 min of **(A, E)** NFκB p65 (Ser536), **(B, F)** Erk1/2 (Thr202/Tyr204, Thr185/Tyr187), **(C, G)** Akt (Ser473), and **(D, H)** mTOR (Ser2448). For comparison results from control *Trpm7^R/R^* neutrophils (open green triangle) were taken from the respective panels above. Data are normalized to protein content and represented as mean ± s.e.m.; n = 3, measured in duplicates; a total number of 5–6 mice were used for each genotype. Statistics: one-way ANOVA *p < 0.05, **p < 0.01, ***p < 0.005. **(I)** Molecular model illustrating the role of TRPM7 in neutrophil chemotaxis and function: in macrophages, LPS-dependent endocytosis of TLR4 and subsequent NFκB activation was shown to depend on TRPM7 (12). We confirm a similar pathway involving TRPM7 channel activity in neutrophils (red dashed arrow). Previously, TRPM7 channel moiety was suggested to be positioned alongside the PI3K signaling axis (red dashed arrow). In neutrophils, PI3K isoforms are activated *via* CXCL8 and CXCR1/2, further triggering the activation of Akt1, NFκB, and mTOR signaling cascades. Alternatively, we propose a model in which TRPM7 kinase is directly or indirectly phosphorylating Akt1, thereby controlling NFκB and mTOR signaling pathways (blue dashed arrow).

To further deliniate the signaling pathways by which TRPM7 channel or kinase alter murine neutrophil function, we analyzed the effect of pharmacologic modulation and genetic TRPM7 kinase inhibition on cellular signaling. Bone marrow derived murine neutrophils from Trpm7^+/+^ and *Trpm7^R/R^* mice were used to analyze the phosphorylation status of NFκB (p65 Ser^536^), ERK1/2 (Thr^202^/Tyr^204^, Thr^185^/Tyr^187^), Akt (Ser^473^), and mTOR (Ser^2448^) upon LPS stimulation. In analogy to human neutrophils, TRPM7 channel and kinase were blocked in neutrophils derived from Trpm7^+/+^ mice using either NS8593 (30 µM) or TG100-115 (20 µM). We observed reduced NFκB (p65 Ser^536^) phosphorylation in NS8593 and in TG100-115 treated cells, whereas NFκB signaling was redusced albeit not significatly altered in *Trpm7^R/R^* neutrophils (**Figure 4A**). We found that neither neutrophils from *Trpm7^R/R^* nor NS8593 or TG100-115 treated Trpm7^+/+^ neutrophils showed significant differences in ERK1/2 signaling (**Figure 4B** **)**. Pharmacologic TRPM7 channel blockade, TRPM7 kinase blockade as well as genetic inactivation of the kinase (*Trpm7^R/R^*) led to significant reduction in Akt/mTOR-dependent signaling (**Figures 4C, D** **)**. Notably, the application of the channel blocker NS8593 resulted in a similar LPS-induced phosphorylation status compared to *Trpm7^R/R^* controls for all signaling proteins, further suggesting that TRPM7 channel blockade might also affect kinase activity. To confirm that the effects of pharmacologic inhibition of the TRPM7 channel or kinase using NS8593 or TG100-115 on the respective signaling proteins was not due to off-target effects, we incubated *Trpm7^R/R^* neutrophils for 30 min with NS8593 or TG100-115 prior to LPS stimulation. Indeed, the pharmacologic blockade of TRPM7 kinase by TG100-115 in *Trpm7^R/R^* neutrophils did not further reduce the phosphorylation status of NFκB (p65 Ser^536^), ERK1/2 (Thr^202^/Tyr^204^, Thr^185^/Tyr^187^), Akt (Ser^473^), or mTOR (Ser^2448^) (**Figures 4E–H**) compared to *Trpm7^R/R^* controls. Similar results were found for application of the channel blocker NS8593 (**Figures 4E–H**).

Together our findings indicate that the TRPM7 kinase regulates neutrophil function *via* activation of Akt/mTOR pathways, while channel activity controls neutrophil activity also through NFκB-dependent pathways, most likely due to providing essential calcium and magnesium (**Figure 4I**).

## Discussion

Excessive neutrophil infiltration accelerates tissue damage due to unrestrained inflammation in pro-inflammatory as well as autoinnmue diseases (26, 27). Therfore, it is crucial to understand the mechanisms that regulate neutrophil function and migration. Previously, TRPM7 was suggested to be involved in CD147-triggered Ca^2+^-induced chemotaxis, adhesion, and invasiveness of a human neutrophil cell line as silencing TRPM7 *via* siRNA led to a reduction of neutrophil adhesion (23). However, the precise mechanism how TRPM7 might be involved in neutrophil recruitment still remains to be explored. Utilizing a heterozygous TRPM7 kinase-deficient mouse model, *Trpm7^+/DK^* (39), it was shown that channel and or kinase activity might affect neutrophil rolling and recruitment (25). Consequently, authors concluded that the channel-kinase TRPM7 could have a protective role in cardiovascular inflammation and fibrosis (25). As in the respective model also the channel activity is reduced (39, 40) it is critical to gain a better understanding of the role of TRPM7 channel and kinase activities in the signaling cascades triggering neutrophil recruitment. We here show that pharmacologic and genetic inhibition of TRPM7 kinase affects human and murine neutrophil function. We further identify a potential underlying molecular mechanism, with Akt1 as novel downstream target of the TRPM7 kinase in neutrophils.

Along with other ion channels, TRPM7 has been linked to migration and motility of various different cell types (41). It has been suggested that TRPM7 channel activity is aiding calcium (Ca^2+^) flickers thus steering fibroblast cell migration (22). We here propose, that in addition to facilitating Ca^2+^ or Mg^2+^ influx, TRPM7 might affect migration and motility *via* its kinase domain. TRPM7 might do so *via* direct phosphorylation of myosin II (29, 42). Recently, a compound with TRPM7 kinase inhibitory activity was identified in a small molecule library screen. TG100-115 was the most potent inhibitor in the screen, significantly decreasing cell migration and invasion of breast cancer cells and inhibiting TRPM7 kinase-dependent myosin IIA phosphorylation (29). Although TG100-115 has been reported to suppress TRPM7 ion channel activity (29), in our hands the kinase inhibitor had no effect on ion channel function. Originally, TG100-115 was shown to inhibit PI3K-γ and -δ (43), to which its broad anti-inflammatory and anti-cancerous as well as cardio-protective effects were attributed (43, 44). However, at least in part, this could also be due to its TRPM7 kinase inhibition. To assess whether the observed effects of TG100-115 are primarily due to PI3K-γ/δ blockade, we applied distinct PI3K-γ/δ inhibitors (IPI, NEM) with similar IC_50_ values (43). While affecting neutrophil chemotaxis in response to a CXCL8 gradient, as well as the production of reactive oxygen species (ROS) upon stimulation with the gram-negative bacterial lipopolysaccharide LPS, PI3K-γ/δ inhibitors did not decrease the LPS-triggered phosphorylation of NFκB, ERK1/2, Akt1, or mTOR. Blockade of TRPM7 kinase using TG100-115, however, affected neutrophil chemotaxis as well as ROS production in response to LPS most likely due to a reduction in Akt/mTOR signaling pathways. Interestingly, TRPM7 inhibition with the channel blocker NS8593 resulted in a similar reduction of Akt/mTOR signaling pathways, culminating in diminished neutrophil chemotaxis and ROS production. It is therefore tempting to speculate that TRPM7 channel blockade also reduces kinase activity and that the Mg^2+^ entering through the channel is required for optimal kinase function. TRPM7 kinase-deficient murine neutrophils also displayed reduced chemotaxis toward a CXCL1 gradient, which was further decreased upon TRPM7 channel blockade using NS8593, suggesting that TRPM7 channel function, at least in part, acts independent of TRPM7 kinase due to ion conductance. Previously, TRPM7 activity has already been implicated alongside PI3 kinase signaling pathways (10, 45, 46), however, as of now the impact of TRPM7 channel *versus* kinase moiety on different PI3K isoforms remains to be established. We here used PI3K isoform specific inhibitors as well as genetic inactivation of TRPM7 kinase to shed light on their interrelationship. In human LPS-treated neutrophils downstream analysis of signaling targets did not reveal a direct functional connection between TRPM7 and PI3K-γ/δ. However, functional analyses of neutrophil chemotaxis and ROS production indicated a potential interdependence. One possible explanation could be that, in CXCL1-triggered chemotaxis as well as LPS-induced ROS production, signaling molecules depending on PI3K-γ/δ pathways cannot be remunerated for, while the LPS-dependent phosphorylation of NFkB, Erk1/2, Akt1, and mTOR was compensated by other PI3K isoforms. Accordingly, TRPM7 kinase might directly or indirectly phosphorylate Akt1 and thereby control complex PI3 kinase signaling pathways while the channel function may indirectly act on PI3 kinase signaling *via* providing essential Ca^2+^ and/or Mg^2+^ for proper kinase activity (**Figure 4I**).

Taken together, we have shown that TRPM7 regulates several neutrophil effector functions including transmigration, extravasation, and ROS production. In addition, we were able to determine that TRPM7 kinase activity leads to Akt/mTOR activation, while the TRPM7 channel moiety also mediates activation of NFκB and ERK1/2. Finally, we demonstrate that TRPM7 and PI3K-γ/δ are not directly linked *via* those signaling pathways. Additional work is needed to further sort out how TRPM7 regulates neutrophil effector functions and how TRPM7 and PI3K-γ/δ cooperate in mediating those neutrophil functions. To finally understand whether TPRM7 kinase activity also affects neutrophil transmigration *in vivo*, we employed a TNF-dependent peritonitis model (47). While this short-term model does not exclude potential effects TRPM7 might have on other cell types, including the migration of other immune cells at later stages, it emphasizes the importance of TRPM7 kinase in regulating neutrophil effector functions and highlights the urgent need for more specific pharmacological inhibitors targeting the TRPM7 kinase moiety.

## Data Availability Statement

The original contributions presented in the study are included in the article/**Supplementary Material**. Further inquiries can be directed to the corresponding author.

## Ethics Statement

The studies involving human participants were reviewed and approved by Ethics committee from the Ludwig-Maximilians-Universität München (Az. 611-15). The patients/participants provided their written informed consent to participate in this study. The animal study was reviewed and approved by District Government of Upper Bavaria, Germany. Written informed consent was obtained from the owners for the participation of their animals in this study.

## Author Contributions

WN conceived and performed experiments, analyzed data, and wrote the manuscript. RI conceived and performed experiments and analyzed data. KH performed experiments and analyzed data. MF, MR, and SR performed experiments. MM provided reagents. IB, TG, and MS provided their expertise and feedback and revised the manuscript. SZ conceived and supervised experiments, analyzed data, and wrote the manuscript. All authors contributed to the article and approved the submitted version.

## Funding

This work was supported by a grant from the Deutsche Forschungsgemeinschaft (DFG) TRR-152 to SZ (P14) and TG (P15), DFG SFB914 to MS (B01).

## Conflict of Interest

The authors declare that the research was conducted in the absence of any commercial or financial relationships that could be construed as a potential conflict of interest.

## Supplementary Material

Supplementary Figure 1
**(A)** Differential blood count of Trpm7^+/+^ and *Trpm7^R/R^* mice. Representative dot plot of the gating schemes of the individual white blood cell types (left panel). Quantification of basal neutrophil, lymphocyte, monocyte, and eosinophil count in whole blood of Trpm7^+/+^ (black, n = 4) and *Trpm7^R/R^* (green, n = 5) mice. Data are presented as 1000 (K) cells/µl and indicate mean ± s.e.m. **(B-D)** Gating schemes obtained using flow cytometry from individual figures as indicated.
