# Supplementary material for: TRPM7 Kinase Is Essential for Neutrophil Recruitment and Function via Regulation of Akt/mTOR Signaling
Source: Front Immunol. 2021 Feb 15;11:606893. doi: 10.3389/fimmu.2020.606893 (PMC7917126; doi:10.3389/fimmu.2020.606893)
Supplement: Supplementary Figure 1 — (A) Differential blood count of Trpm7+/+ and Trpm7R/R mice. Representative dot plot of the gating schemes of the individual white blood cell types (left panel). Quantification of basal neutrophil, lymphocyte, monocyte, and eosinophil count in whole blood of Trpm7+/+ (black, n = 4) and Trpm7R/R (green, n = 5) mice. Data are presented as 1000 (K) cells/µl and indicate mean ± s.e.m. (B-D) Gating schemes obtained using flow cytometry from individual figures as indicated. [file Image_1.pdf]

frontiers

Supplementary Information

TRPM7 Kinase is essential for Neutrophil Recruitment and Function *via* Regulation of Akt/mTOR Signaling

Wiebke Nadolni[1], Roland Immler[2], Kilian Hoelting[1], Marco Fraticelli[1], Myriam Ripphahn[2], Simone Rothmiller[3], Masayuki Matsushita[4], Ingrid Boekhoff[1], Thomas Gudermann[1], Markus Sperandio[2] and Susanna Zierler[1,5*]

[1]Walther Straub Institute of Pharmacology and Toxicology, Ludwig-Maximilians-Universität München, Munich, Germany
[2]Walter Brendel Centre of Experimental Medicine, Biomedical Center, Institute of Cardiovascular Physiology and Pathophysiology, Ludwig-Maximilians-Universität München, Planegg-Martinsried, Germany
[3]Bundeswehr Institute of Pharmacology and Toxicology, Munich, Germany
[4]Department of Molecular and Cellular Physiology, Graduate School of Medicine, University of the Ryukyus, 207 Uehara, Okinawa 903-0215, Japan
[5]Institute of Pharmacology, Johannes Kepler University Linz, Linz, Austria

Correspondence:
Correspondence should be addressed to: susanna.zierler@lrz.uni-muenchen.de or susanna.zierler@jku.at

Content: Supplementary Figure 1 (Figure S1)

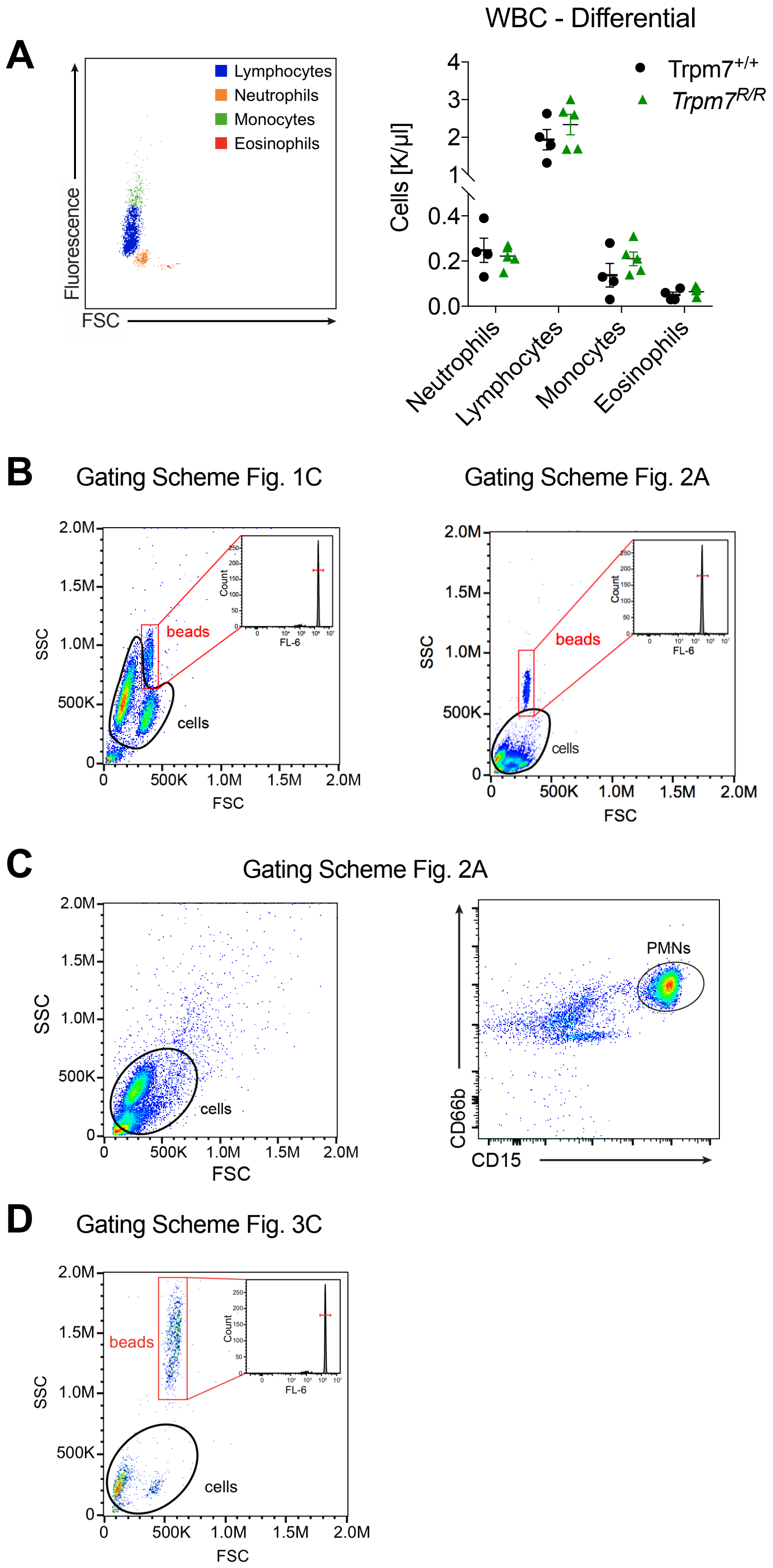

Figure S 1. (**A**) Differential blood count of Trpm7$^{+/+}$ and *Trpm7*$^{R/R}$ mice. Representative dot plot of the gating schemes of the individual white blood cell types (left panel). Quantification of basal neutrophil, lymphocyte, monocyte, and eosinophil count in whole blood of Trpm7$^{+/+}$ (black, n = 4) and *Trpm7*$^{R/R}$ (green, n = 5) mice. Data are presented as 1000 (K) cells/µl and indicate mean ± s.e.m. **(B-D)** Gating schemes obtained using flow cytometry from individual figures as indicated.
