# Supplementary figures and images for: TRPM7 Kinase Is Essential for Neutrophil Recruitment and Function via Regulation of Akt/mTOR Signaling
Source: Front Immunol. 2021 Feb 15;11:606893. doi: 10.3389/fimmu.2020.606893 (PMC7917126; doi:10.3389/fimmu.2020.606893)

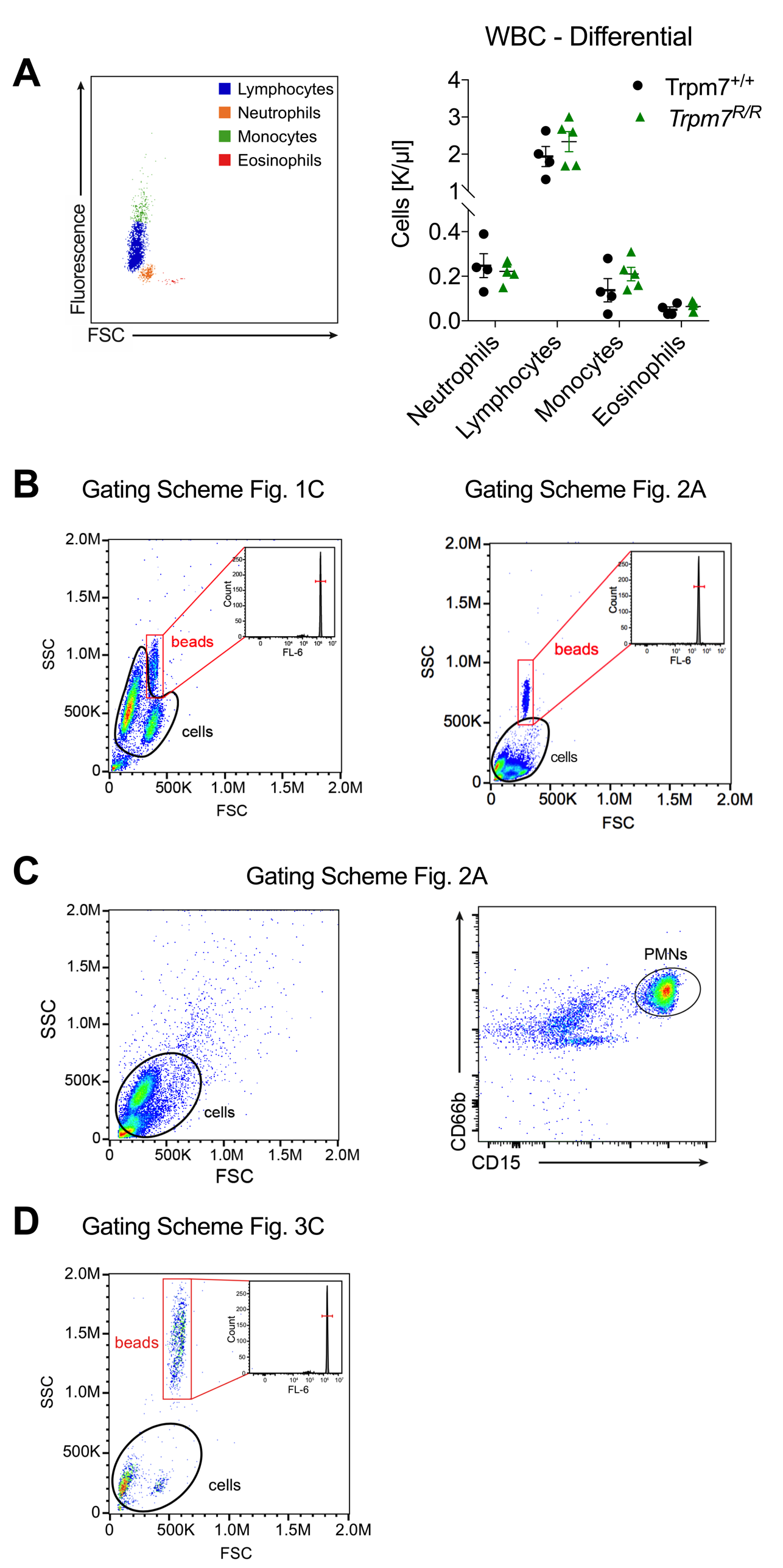

Supplement: Supplementary file 2 [file Image_2.tiff]
